# Gastric Adenocarcinoma Presenting as a Rheumatoid Factor and Anti-cyclic Citrullinated Protein Antibody-Positive Polyarthritis: A Case Report and Review of Literature

**DOI:** 10.3389/fmed.2021.627004

**Published:** 2021-05-24

**Authors:** Manuel Silvério-António, Federica Parlato, Patrícia Martins, Nikita Khmelinskii, Sandra Braz, João Eurico Fonseca, Joaquim Polido-Pereira

**Affiliations:** ^1^Rheumatology Department, Hospital de Santa Maria, Centro Hospitalar Universitário Lisboa Norte, Lisbon Academic Medical Centre, Lisbon, Portugal; ^2^Rheumatology Research Unit, Faculdade de Medicina, Instituto de Medicina Molecular, Universidade de Lisboa, Lisbon, Portugal; ^3^Medicina 2 Department, University Hospital Center of Lisbon North, Lisbon, Portugal

**Keywords:** paraneoplastic syndrome, paraneoplastic arthritis, seropositive arthritis, rheumatoid arthritis, gastric cancer

## Abstract

A 64-year-old male presented with a 6-month history of symmetric polyarthritis involving proximal interphalangeal joints and metacarpophalangeal joints of the hands, wrists, and ankles. Associated symptoms included vomiting, progressive fatigue, and weight loss. Laboratory results showed microcytic anemia, leukocytosis, thrombocytosis, elevated C-reactive protein and erythrocyte sedimentation rate, and rheumatoid factor (RF) and anti-cyclic citrullinated protein (ACPA) antibody positivity. Joints radiographs were normal, without erosions. Upper endoscopy and gastric endoscopic ultrasonography showed a gastric adenocarcinoma with lymphatic involvement. Intraoperatively, peritoneal carcinomatosis was documented, and the patient started palliative chemotherapy. A paraneoplastic seropositive arthritis was assumed, and treatment with low-dose prednisolone and hydroxychloroquine was started. Arthritis remission was achieved and sustained up to 18 months of follow-up, although gastric cancer progression was documented. We describe a unique phenotype of paraneoplastic arthritis (PA) presenting as a seropositive (RF and ACPA positivity) rheumatoid arthritis (RA) with a good response to both low dose corticosteroids and hydroxychloroquine therapy. We also review the literature of PA, mostly the RA-*like* pattern, and the association between PA and ACPA positivity. This case highlights the importance of considering underlying cancer in elderly male patients, presenting with polyarthritis and systemic symptoms, even in those with ACPA-positive RA-*like* arthritis.

## Introduction

Paraneoplastic syndromes are manifestations of a malignant disease that are not directly caused by the tumor itself and occur distant from the primary tumor or metastasis. Temporal relationship is the main critical issue to identify paraneoplastic syndromes, and as a rule, the manifestations must occur during a malignant disease or precede it by no longer than 2 years (1–3). The best evidence for the association between the clinical manifestations and the malignant disease is established retrospectively when the symptoms improve or completely remit with the treatment of the underlining neoplasm (1, 2). A spectrum of rheumatologic manifestations can result from a paraneoplastic process and are present in about 7–10% of patients with cancer, including arthritis, lupus-like syndromes, inflammatory myopathies, vasculitis, and hypertrophic osteoarthropathy (1, 3–5).

Paraneoplastic arthritis (PA) can present similarly to other inflammatory arthritis, and some features are used to distinguish it from those diseases. It affects more often elderly men (male/female ratio of 1.7:1 and a median age of onset of 54 years) and was classically described as a seronegative and non-erosive asymmetrical oligo-polyarthritis involving most frequently the knees, ankles, wrists, and hands (5–7). Recently, a symmetric polyarthritis indistinguishable from rheumatoid arthritis (RA) as a presenting feature of PA has been documented, and isolated case reports of PA with rheumatoid factor (RF) and/or anti-cyclic citrullinated peptide antibody (ACPA) positivity have been published (4, 8–11). These data suggest that arthritis' pattern and serology may not be entirely reliable in the identification of possible cases of PA (4). In this case-based review, we report a gastric adenocarcinoma presenting as RA with RF and ACPA positivity, and we review the literature of PA, mostly the seropositive pattern.

## Case Report

A 64-year-old male presented to the emergency department with a 6-month history of gradual onset of joint pain, swelling, and morning stiffness involving the wrists, metacarpophalangeal (MCP) joints, proximal interphalangeal (PIP) joints, and ankles. In addition, he complained of occasional vomiting, anorexia, progressive fatigue, and unintentional 12 kg of weight loss. His arthralgia was progressive and debilitating, and had a poor response to non-steroidal anti-inflammatory drugs (NSAIDs), acetaminophen, and opioid analgesics (tramadol 200 mg daily).

His past medical history and medication were irrelevant besides being an ex-smoker (10 pack-years, stopped 10 years ago). He had a family history of gastrointestinal and pulmonary malignancies. He denied any oral ulcers, dry eyes, rash, fever, night sweats, gastrointestinal bleeding, or other complaints. On physical examination, arthritis of the wrists, MCP joints, PIP joints, and ankles, and a positive metatarsal squeeze test were noted. The rest of the examination was unremarkable.

The initial laboratory investigations uncovered a microcytic and hypochromic anemia (hemoglobin 7.9 g/dl), leukocytosis (12.7 × 10^9^/L), thrombocytosis (904 × 10^9^/L), and elevation of C-reactive protein (CRP; 17.3 mg/dl) and erythrocyte sedimentation rate (ESR; 94 mm/h). Renal and liver profiles were normal, and an infection screen was negative. An anemia investigation showed low serum iron (9.6 μg/dl) and transferrin saturation (4%), and normal ferritin (74.1 ng/dl), cyanocobalamin (465 pg/ml), and folate (5 ng/ml) levels. Radiographic evaluation of the affected joints and chest was unremarkable. The RF and ACPA were significantly elevated at 215 IU/ml (0–14 IU/ml) and 156.9 IU/ml (<20 IU/ml), respectively.

**Figure 1:**
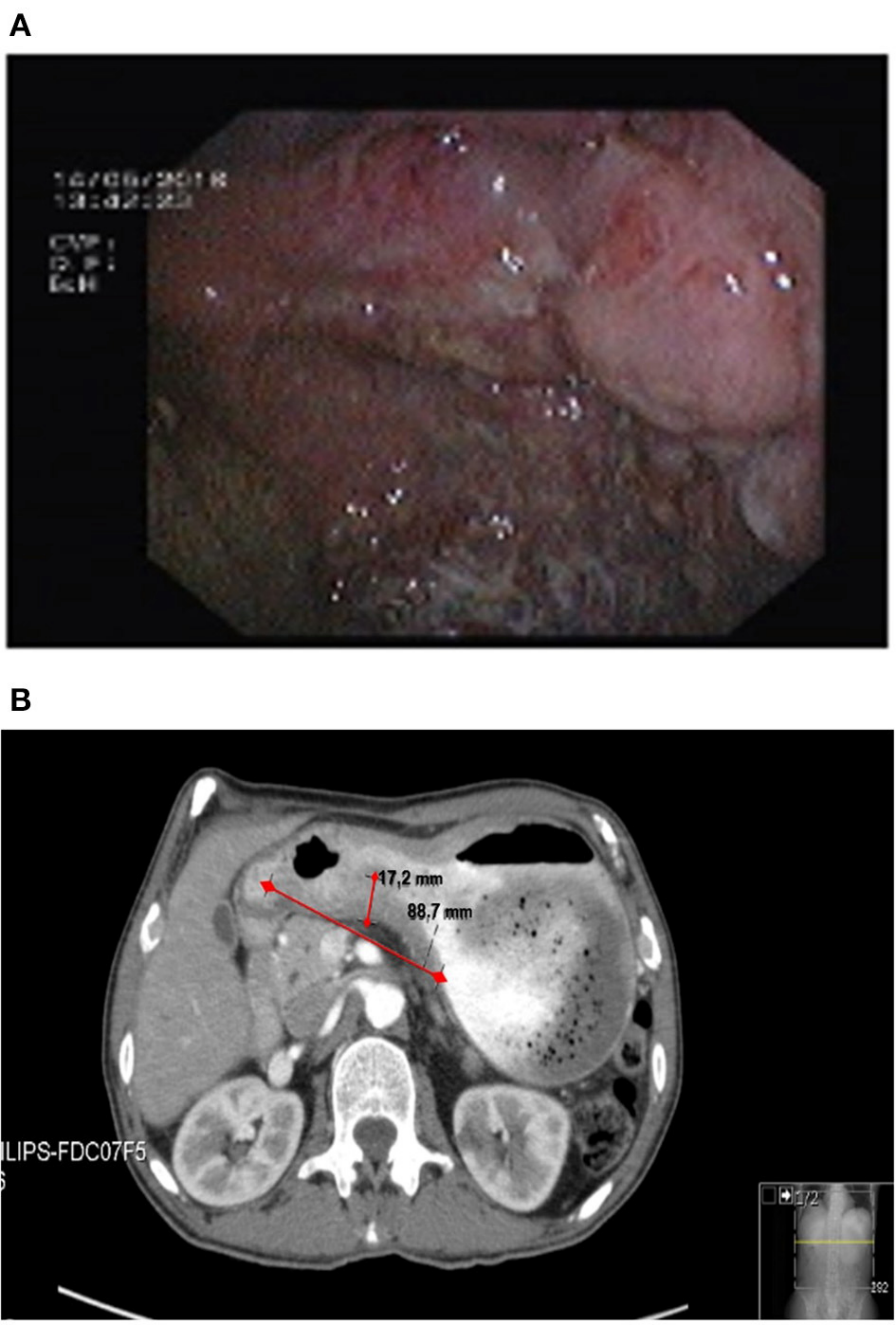
Gastric mass on **(A)** upper gastrointestinal endoscopy and **(B)** abdomen computed tomography (red arrow).

Upper gastrointestinal endoscopy exhibited on the lesser curvature and antrum of the stomach a hard and friable mass with areas of necrosis that conditioned luminal stenosis (Figure 1A). Endoscopy ultrasound of the upper gastrointestinal tract revealed a mass extending 17 mm to the serosa layer with regional involvement of four lymph nodes. Endoscopy biopsy specimens showed a gastric adenocarcinoma with poor differentiation. Contrast-enhanced chest–abdomen–pelvis computed tomography confirmed the gastric mass with no evidence of other adenopathy or distant metastasis (Figure 1B), and the tumor was rated T3N2M0, G3. Chest evaluation showed slight changes compatible with emphysema.

**Figure 2:**
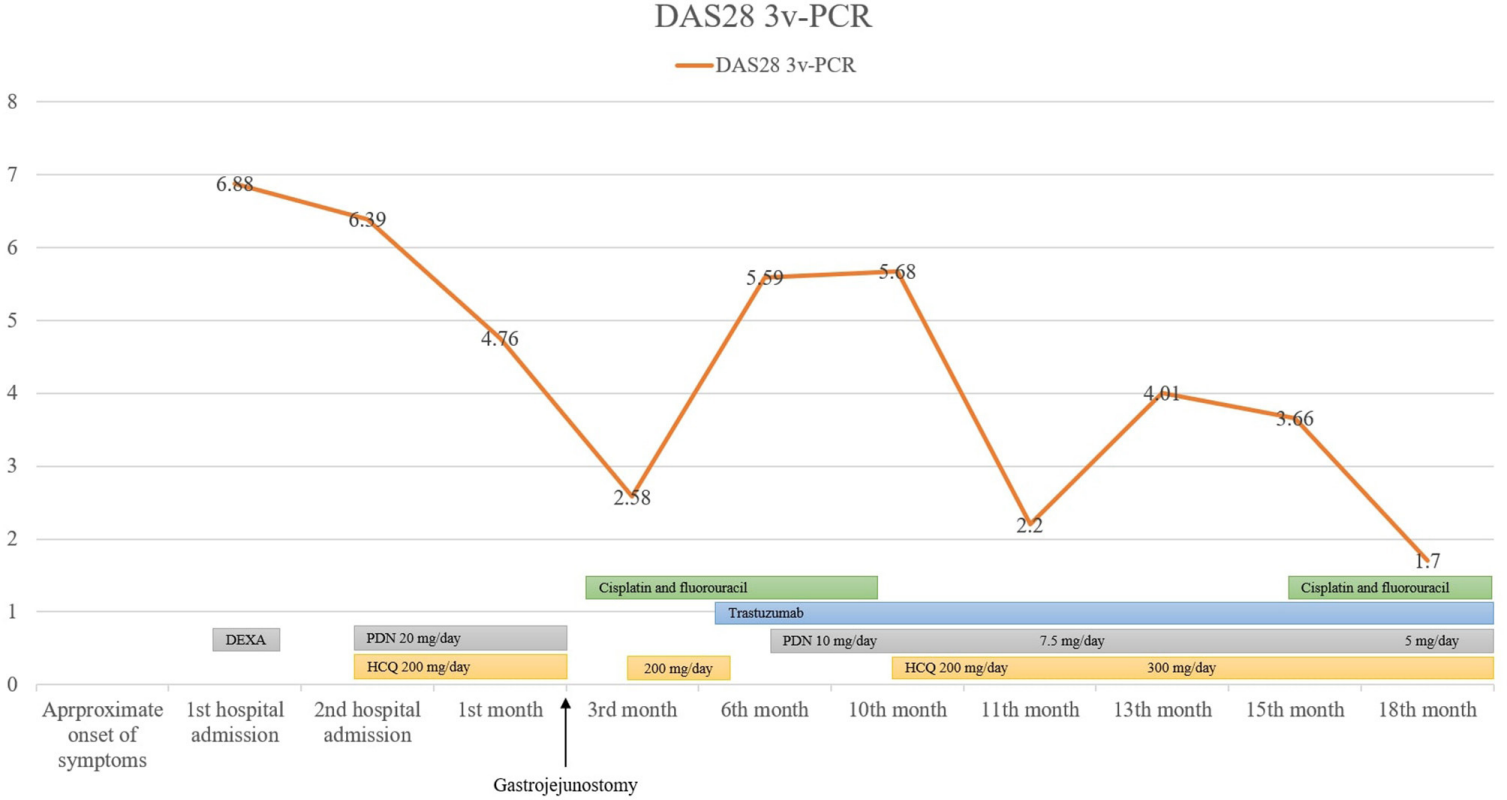
Timeline disease activity of arthritis and relation with chemotherapy drugs, corticosteroids, and hydroxychloroquine. DAS28 3v-PCR, Disease Activity Score for 28 joints with three variables (tender joint count, swollen joint count, and C-reactive protein); DEXA, dexamethasone; HCQ, hydroxychloroquine; PDN, prednisolone.

During the hospitalization, while the malignancy workout was ongoing, polyarthritis was treated with a short course of corticosteroids (dexamethasone 5 mg daily) with good clinical response, and the dose was quickly tapered. However, a few weeks later, his symptoms recurred, and he was readmitted due to severe polyarthritis with disabling gait. Corticosteroid therapy was reintroduced (prednisolone 20 mg daily), and hydroxychloroquine (200 mg daily) was also added. However, full adherence to the therapeutic regimen was only possible after a few months and only then was a complete resolution of polyarthritis achieved (Figure 2).

He was referred to oncology and proposed for gastrectomy. During the laparotomy procedure, extensive peritoneal carcinomatosis was detected, whereby he underwent gastrojejunostomy. The patient started palliative chemotherapy with cisplatin and fluorouracil, and after HER2 overexpression was documented, trastuzumab was added. Cisplatin and fluorouracil were stopped after 8 months, but cancer progression was documented and were reintroduced later on, with little effect on arthritis control. After 18 months of follow-up, he remained alive, with sustained arthritis remission for over 9 months (documented clinically and using ultrasound) and without radiological detectable erosions, under treatment with low-dose prednisolone (<7.5 mg daily) and hydroxychloroquine (300 mg daily), despite the absence of a curative cancer treatment.

## Discussion

Overall, patients with RA are at an increased risk of cancer compared with the general population. However, gastric cancer has a lower incidence in RA patients, which can be explained by the increased use of NSAIDs in this population (12–15). The incidence of RA in patients with gastric cancer is not known.

A wide variety of solid and hematological malignancies has been associated with PA, with lung adenocarcinoma being the most frequent (3, 9). Thus, far, only four patients with PA were reported having gastric tumors, none with a seropositive RA-like pattern (3, 16–18).

Based on the review of case reports, PA was traditionally described as a seronegative asymmetric oligoarthritis with predominant involvement of the lower limb joints (Table 1). However, a symmetric polyarthritis indistinguishable from RA is now recognized to be the most frequent pattern, in a few cases associated with RF or ACPA.

**Table 1:** Traditional characteristics of paraneoplastic arthritis [adapted from Stummvoll et al. (5), Larson et al. (8), Caldwell and McCallum (19), and Pfitzenmeyer et al. (20)].

1. Close temporal relationship (12 months) between the onset of arthritis and malignancy
2. Older age
3. Joint involvement in an asymmetric distribution
4. Predominance of lower extremity involvement
5. Explosive onset
6. Absence of family history of rheumatic disease
7. Absence of rheumatoid nodules
8. Absence of characteristic radiographic lesions of RA
9. Absence of RF
10. Non-specific histopathologic appearance of the synovial lining

*RA, rheumatoid arthritis; RF, rheumatoid factor*.

Classic PA features were detailed by Kisacik et al. (7) by comparing patients with PA (*n* = 65) with patients diagnosed with early RA (*n* = 50). In this study, the PA group was defined by using previously described traditional features, and the inclusion criteria were rapid disease onset and symmetric or asymmetric disease mostly affecting the lower extremities and usually sparing the small joints of the hands. Consequently, patients with RA-like arthritis were excluded from the PA group, which originated an unintentional bias in the description of PA characteristics.

An earlier study by Morel et al. better characterized this type of arthritis, in a case series of 26 patients with PA, using a less restrictive arthritis pattern—oligo-polyarthritis evolving for a maximum of 2 years before cancer diagnosis (3). Despite confirming most of the traditional features, such as the absence of RF and radiologic lesions, they concluded that the majority of patients displayed features of RA, being symmetric polyarthritis, involving wrists and hands, the most frequent articular pattern (85%). Therefore, they proposed a new group of characteristic features for the diagnosis of PA (Table 2).

**Table 2:** New characteristic features proposed for the diagnosis of paraneoplastic arthritis [adapted from Morel et al. (3)].

1. Average time between arthritis and neoplasia diagnosis <6 months
2. Men age >50 years
3. Polyarthritis (symmetric or asymmetric)
4. Absence of rheumatoid nodules
5. Absence of erosions on radiography
6. Absence of RF
7. Degradation of general health status
8. High CRP level
9. Regression of arthritis after specific anti-tumoral therapy

*CRP, C-reactive protein; RF, rheumatoid factor*.

A review by Watson et al. also included several patients with PA with symmetric distribution and involvement of the wrists, small joints of the hands, and knees, resembling RA. Contrary to previous reports, some of these patients were positive for both RF and ACPA (9).

Our patient presented with an RA-like pattern and high titer for both RF and ACPA meeting the 2010 American College of Rheumatology/European League Against Rheumatism classification criteria for RA. To the best of our knowledge, this is the sixth case described in the literature classifiable as a PA with RF and ACPA positivity (Table 3). Of interest, four of these patients also had an RA-like pattern, with erosive arthritis present in one of them. Most of the patients responded well to low-dose corticosteroid therapy, but due to the advanced stage of the malignancies, tumor therapy was curative in a single patient, and only in this case, a complete resolution of the PA occurred. None of the reported cases mentions the prescription of disease-modifying anti-rheumatic drugs (DMARDs), which may be explained by the concomitant chemotherapy or predictable poor general condition of a patient with advanced neoplastic disease.

**Table 3:** Characteristic features of published cases of RF and ACPA-positive paraneoplastic arthritis.

**Reference**	**Age/sex**	**Type of malignancy**	**Duration of symptoms preceding cancer diagnosis (months)**	**Family history of cancer**	**Arthritis location and pattern**	**Onset**	**Anemia**	**ESR (mm/h)/ CPR (mg/dl)**	**RF (IU/ml) /ACPA (IU/ml)**	**Joint radiographs**	**Response to NSAIDs**	**Response to corticosteroids**	**Cancer treatment used (response or evolution)**
Kumar et al. (11)	58/M	Pancreatic cancer	2	N.M.	No arthritis; arthralgia involving hands, wrists, elbows, shoulders, lower back, and neck; asymmetric and intermittent	Gradual	Yes	++/N.M.	+++/++	N.E.	N.M.	Yes	None (died)
Raja et al. (10)	40/M	Lymphomatoid granulomatosis	3–4	No	Wrists, knees, and ankles; symmetric	Gradual	No	–/+	++/++	N.E.	N.M.	Yes (partial)	None (died)
Larson et al. (8)	45/F	Lung adenocarcinoma	3	No	PIP, MCP, elbows, and knees; symmetric	N.M.	No	–/+	++/+	N.E.	No	Yes (partial)	None (died)
Handy et al. (4)	61/F	T cell lymphoblastic leukemia	1–2	No	MCP, wrists, knees, and ankles; symmetric	Acute	Yes	+++/++	+++/++	Erosions	No	No	Hyper-CVAD CMT (refractory)
Watson et al. (9)	80/F	Breast papillary cancer	1	N.M.	Shoulder arthritis; arthralgia involving wrists, shoulders, and knees; asymmetric and migratory	Acute	Yes	–/++	+/+	N.E.	No	Yes	CMT (N.M) and RT (remission)
Present case	64/M	Gastric adenocarcinoma	6	Yes (GI and lung)	PIP, MCP, wrists, and ankles; symmetric	Gradual	Yes	++/+++	++/++	N.E.	No	Yes	5-FU and CIS CMT (not curative)

*CPR, C-reactive protein; ESR, erythrocyte sedimentation rate; ACPA, anti-cyclic citrullinated peptide antibodies; RF, rheumatoid factor; NSAIDs, non-steroidal anti-inflammatory drugs; F, female; M, male; GI, gastrointestinal; MCP, metacarpophalangeal joints; PIP, proximal interphalangeal joints; 5-FU, fluorouracil; CIS, cisplatin; hyper CVAD, cyclophosphamide, vincristine, doxorubicin, and dexamethasone; CMT, chemotherapy; RT, radiotherapy; N.E., no erosions; N.M., not mentioned*.

*ESR: – if <30, + if ≥30, and <60, ++ if ≥60 and <100, +++ if ≥100*.

*CPR: + if ≥0.5 and <5.0, ++ if ≥5.0 and <15.0, +++ if ≥15.0*.

*RF titer: + if ≥14 and <100, ++ if ≥100 and <300, +++ if ≥300*.

*ACPA: + if ≥20 and <100, ++ if ≥100 and <300, +++ if ≥300*.

Despite the fact that PA was classically described as refractory to corticosteroids and NSAIDs, the study by Morel et al. showed some contradictory information. NSAIDs and corticosteroids were effective, respectively, in 45 and 91% of the patients, but without obtaining a sustained remission. DMARDs (sulfasalazine and methotrexate) were only effective in one out of five treated patients.

Two studies analyzed a series of patients with paraneoplastic rheumatologic syndromes and showed that the majority of PA were ACPA negative (7, 21). ACPA have similar sensitivity as RF to RA but with a higher specificity (90–95%), thus, allowing more accurate diagnosis of RA, especially in cases of early undifferentiated arthritis (8, 9). However, this test is not 100% specific, and ACPA positivity has been reported in a variety of diseases (21–25). As this case highlights, the presence of ACPA in the context of a polyarthritis should not exclude automatically other diagnostic entities, particularly in elderly patients with consumptive symptoms. Overlooking this might delay the diagnosis and treatment of underlying cancer (8, 9).

ACPAs are formed against citrullinated antigens that appear after enzyme-catalyzed peptide transformation by peptidylarginine deiminase (PAD) enzymes. In RA, these antigens are found in inflammatory synovial membranes, and ACPAs have been shown to perpetuate joint inflammation and correlate with more aggressive disease (26). In hematological and solid tumors, PAD enzymes are upregulated and have been implicated in the carcinogenesis process (27). In 2014, Handy et al. proposed a possible association between ACPA positivity and PA (4). They suggested that ACPA could appear due to the overexpression of PAD enzymes in malignant diseases, generating citrullinated peptides, which are then exposed to the immune system. However, this hypothesis does not explain all PA cases (as most are ACPA negative) nor offers a complete mechanistic elucidation of the PA phenomena.

In the present case, the onset of a symmetric polyarthritis in an elderly man, associated with constitutional symptoms and iron deficiency anemia led us to an additional workup. In our opinion, the characteristic features proposed by Morel et al. (3) (Table 2) are more representative of the full spectrum of PA and should be preferred in clinical practice.

Cancer progression was seen in our patient and was accompanied by flares of polyarthritis, despite ongoing chemotherapy. We should note that some chemotherapy drugs can improve RA clinical manifestations, namely, cisplatin and fluorouracil, which were used in this patient (28, 29). However, in this case, chemotherapy drugs did only have a modest effect, and arthritis remission was only possible when full adherence to hydroxychloroquine and prednisolone was achieved. Since the effect of hydroxychloroquine in monotherapy in RA is quite modest when compared with other classic DMARDs, especially in double seropositive RA, it is probable that in our patient, the arthritis remission was achieved due to the combination of hydroxychloroquine, prednisolone, and chemotherapy (30).

Accordingly, we believe our case represents a seropositive PA, being the first case related to gastric malignancy. Unfortunately, the staging of our patients' malignant disease determined palliative surgery and chemotherapy, and the likelihood of curative treatment is very low. We cannot, thus, conclude if arthritis would remit with the treatment of underlying cancer.

In conclusion, our case illustrates that physicians should be aware and rule out cancer in an ACPA-positive RA-like polyarthritis in patients with constitutional symptoms and/or with laboratory features showing severe anemia, thrombocytosis, and very high CRP.

## Data Availability Statement

The original contributions presented in the study are included in the article/supplementary material, further inquiries can be directed to the corresponding author/s.

## Ethics Statement

Ethical review and approval was not required for the study on human participants in accordance with the local legislation and institutional requirements. The patients/participants provided their written informed consent to participate in this study.

## Author Contributions

MS-A contributed to the manuscript conception, design, and literature review. MS-A, FP, PM, NK, SB, JF, and JP-P contributed to the manuscript preparation and critical review. All authors have read and approved the final version of the manuscript.

## Conflict of Interest

The authors declare that the research was conducted in the absence of any commercial or financial relationships that could be construed as a potential conflict of interest.
